# COGNITIVE FUNCTION AND HELP-SEEKING AMONG US CHINESE OLDER ADULTS WITH ELDER MISTREATMENT

**DOI:** 10.1093/geroni/igac059.1507

**Published:** 2022-12-20

**Authors:** Ying-Yu Chao, An-Yun Yeh, Peijia Zha, XinQi Dong

**Affiliations:** Rutgers, the State University of New Jersey, Newark, New Jersey, United States; College of Staten Island CUNY, New York, New York, United States; Rutgers, the State University of New Jersey, Newark, New Jersey, United States; Rutgers, New Brunswick, New Jersey, United States

## Abstract

**Purpose:**

Elder mistreatment (EM) in immigrants with cognitive decline is an understudied public health problem. Cognitive function plays an important role in individuals’ ability to seek help. This study aimed to examine the associations between cognitive function and help-seeking among U.S. Chinese older adults reported EM.

**Methods:**

Data were from the Population Study of Chinese Elderly in Chicago (PINE). Five instruments were used to measure cognitive function, including the Mini-Mental State Examination, East Boston Memory Test Immediate Recall and Delayed Recall, Digit Span Backwards, and Symbol Digit Modalities Test. Informal/formal help-seeking intentions and behaviors were measured. Descriptive statistics, linear regression, and logistic regression were performed.

**Results:**

A total of 450 participants reported EM. Most victims sought help from informal sources (53.48%), followed by any sources (11.42%) and formal sources (3.34%). About one-third of participants did not seek any help. Higher episodic memory was associated with an increase in help-seeking intentions among Chinese older adults with financial mistreatment (p < .05) and poly-victimization (p < .01). Lower working memory was associated with an increase in help-seeking intentions among those with caregiver neglect (p < .001). Compared to not seeking help, higher executive function was associated with a higher likelihood to seek help from any sources among Chinese older adults with psychological mistreatment (p < .05). Conclusion/implication: This study highlights the associations between cognitive function and help-seeking among this underserved population. Culturally tailored interventions are suggested to promote help-seeking for different types of cognitive impairment among Chinese older adults with EM.

